# The electronic health record as a teaching and learning tool in residency training: A scoping review

**DOI:** 10.12688/mep.20947.1

**Published:** 2025-05-07

**Authors:** Anna Rubin, Reema Kola, Catherine Varnum, Parvinder Khurana, Hari Magge, Zareen Zaidi

**Affiliations:** 1 Department of Medicine, The George Washington University School of Medicine and Health Sciences, WASHINGTON, DC, 20037, USA; 2 Medical student, The George Washington University School of Medicine and Health Sciences, Washington, DC, USA

**Keywords:** Electronic health record; Graduate medical education; Practice-based learning and improvement; Systems-based practice; Teaching

## Abstract

**Background:**

While electronic health records (EHRs) are a critical tool for clinical care in academic medical centers, there is a gap in the literature regarding utilization of EHRs for teaching and learning, outside of descriptions on EHR training or use for quality improvement projects. The research question for this scoping review is: How is the EHR purposefully used in educational interventions for teaching and learning in graduate medical education?

**Methods:**

We searched all publications in PubMed, SCOPUS, CINAHL, ERIC, Cochrane and MedEdPORTAL databases from 2010 to 2022 that met our search criteria and MeSH terms. Using Arksey and O'Malley's framework, the abstracts were reviewed by three team members, and the full text was reviewed by all authors using a standardized data extraction sheet.

**Results:**

Of the 2431 records, 19 met inclusion criteria and were reviewed in full. All studies were set in North America, 79% at teaching hospitals, and 42% in Internal Medicine programs. The EHR was used purposefully to improve learning in sepsis, diabetes, asthma, critical care, post- and peri-operative care, and it was used to provide feedback about clinical performance, test utilization, costs of care, and errors. The educational interventions included simulation, direct feedback using EHR data, and workshops with pre-post knowledge and skills testing.

**Conclusions:**

This scoping review identifies how the EHR is being used as a teaching and learning tool for trainees. There is a need for more purposeful use of the EHR in designing educational interventions for residents.

## Introduction

Electronic Health Records (EHRs) have become an integral part of clinical care in the United States with 78% out-patient practices and 96% hospitals adopting a certified EHR ^1^. This is a drastic increase, nearly 4-fold since 2011. EHRs are also widely adopted by academic medical centers and have become a critical tool for health care in training programs ^2^. As EHRs become the standard of care in US healthcare practice, new approaches to integrating this technology into the teaching of medical students and resident physicians are necessary. The Accreditation Council for Graduate Medical Education (ACGME) Milestones encourage digital health proficiency as trainees also learn clinical care while ‘managing’ patients via the EHR ^3, 4^. EHRs have the potential for not only facilitating and streamlining patient care, but they also hold the promise of providing training in all domains of core competencies ^5^. Because of the rapid adoption of the EHR, there is a lag between its use for clinical care and its mobilization as a teaching tool to enhance medical education ^6^. While there are descriptions of training residents on how to use EHRs efficiently or how to use EHR features for quality improvement projects, there is a gap in the literature about their use in purposefully designed educational interventions for teaching and learning. The research question for this scoping review is: How is the EHR purposefully used in educational interventions for teaching and learning in graduate medical education?

## Methods

We conducted a scoping review to examine the existing literature to identify how the EHR is being purposefully used as a teaching and learning tool in graduate medical education (residency training). We used the Arksey and O’Malley framework ^7, 8^ to conduct the scoping review, and the Preferred Reporting Items for Systematic Reviews and Meta-analyses Extension for Scoping Reviews (PRISMA-ScR) guided the research design and reporting ^9^.

### Step 1: Identifying the research question

Following the first step of the scoping review framework, all authors discussed their conceptualizations of how the EHR can be used as a teaching and learning tool and the need to study the effectiveness through purposefully designed educational intervention studies. The discussion led to our final research question: “How is the EHR purposefully used in educational interventions for teaching and learning in graduate medical education?”

### Step 2: Identifying relevant studies

The research team collaborated with a research librarian to develop a search strategy and identify articles on the EHR in residency education. Using keywords and MeSH terms related to the research question (Appendix 1, extended data) a search was conducted in PubMed, Medline, Scopus, CINAHL, Cochrane and MedEdPORTAL databases between 2010 to November 2022. Search terms were refined iteratively. The last search was performed on November 5, 2022. The search results were downloaded to Covidence®, a proprietary systematic review software tool (Rayyan ® is an alternate free platform that scholars can consider using).

### Step 3: Study selection

***Step 3.1: Inclusion and exclusion criteria***

We included all full text papers published in the English language that described interventions where the EHR was used with trainees at any graduate medical education program globally and included a description of a *purposeful design for an educational intervention* to assess the use of EHR for teaching and learning in trainees. We encountered multiple studies in the literature that only used the EHR for data collection or that used an EHR intervention to improve a clinical outcome or quality metric but did not subsequently examine the residents’ learning experience or sustained behavior change as a result of the intervention. Overall, we excluded non-English studies, studies that focused only on teaching trainees how to use the EHR or improve efficiency of use, studies that aimed at increasing only the documentation of a clinical outcome or metric without follow up on trainee learning, and EHR interventions that were reminders for best practices.

***Step 3.2: Selection process***

Study team members AR, RK, HM and ZZ screened titles and abstracts uploaded to Covidence (n=3962) for inclusion; after removal of duplicate studies the n=2431. After the abstract screening, 43 studies met our inclusion criteria. Three reviewers (AR, RK and ZZ) then independently screened full-text articles (n=43) to further exclude studies that did not meet the aim of the research question. All conflicts were resolved in team meetings through discussion with study team members. Ultimately, nineteen studies met our inclusion criteria and answered our research question about how the EHR is purposefully used for teaching and learning in residency education. Figure 1 illustrates the study selection process, see extended data.

### Step 4: Charting the data

The full-text articles were independently reviewed by five study authors. RK, CV and PK contributed to the data extraction and charting process. A data extraction form was developed a priori and pilot-tested and refined further. The information extracted from the papers were authors’ names, publication data, country, purpose of the study, trainee level and field of training, type of educational intervention, measured outcomes, and the effects of intervention on learning outcomes. The data was compared and discussed during team meetings to achieve consensus.

### Step 5: Collating, summarizing and reporting the results

The scoping review included a descriptive analysis of the selected studies, as each study had its own individual context and the results would not be amenable to statistical analysis. For the descriptive analysis we used content and thematic analysis ^10^. The characteristics of the study are shown in Table 1.

**Table 1.** Characteristics of studies meeting the inclusion criteria.

Authors	Purpose	Study Type	Intervention(s)	Participants & setting	Outcome Assessment(s)	Results	Conclusion
Lee *et al.* ^11^	To introduce the purpose and value of the ICD-10-CM to residents through a simulated EHR experience	Before and after	1. Three different 50-min team-based sessions with simulated EHR clinical vignettes with assigned tasks 2. Pre-session article review (60 min)	Internal Medicine residents / Outpatient practice in a university setting: Temple University Hospital	1. Multiple Choice Exams (before and after) 2. Survey/ Questionnaire of learners before and after	The number of correctly answered questions increased from 6.05 in the pre- to 6.55 post-intervention. 88% found the exercise useful, 94% committed to appropriate ICD-10-CM documentation following the module, and 76% stated that they would like similar exercises.	Understanding ICD-10-CM coding empowers residents to document with greater specificity and accuracy as they advance in their practice.
Smith *et al.* ^12^	To determine if the teaching EHR tool improves learners’ ability to enter an evidence-based, comprehensive initial care plan for critically ill patients	Before and after	One time 2-hour session consisting of 3 modules with de-identified patient data with assigned task (i.e. to place admission orders)	Internal Medicine and Medicine-Pediatrics residents / Intensive Care Unit in a university setting: Indiana University School of Medicine	1. Data from learner’s performance during the simulations 2. Survey/ Questionnaire of learners before and after	Average diagnostic accuracy improved from 44.4% to 80.6% among residents who completed all 3 modules. 85% of residents reported the EHR tool was an effective form of teaching and 89% would use more modules if provided. Resident comfort with caring for critically ill patients improved after the session but was not statistically significant.	The EHR educational tool is a practical and positively received approach to enhancing residents' training and evaluation in core clinical care and patient management, despite duty hour constraints.
Greene *et al.* ^13^	To determine if providing residents with objective measures of their individual adherence to evidence-based post-operative nausea/vomiting (PONV) management protocols improves their compliance with standardized treatment methods	Before and after, and prospective cohort study	1. One 15-min lecture of PONV risk factors and guidelines 2. EHR data report x 2 of individual PONV guideline compliance/ performance emailed to residents x 2	Anesthesiology residents / Peri-operative management in a university setting: University of Washington School of Medicine, Seattle, WA	1. EHR data before and after educational intervention 2. Survey/ Questionnaire of learners after	After feedback, patients received a significantly higher number of post-operative nausea and vomiting prophylactic treatments (p=0.001, mean 1.35 vs. 1.99). PONV management protocol compliance increased from 38% to 73% (p<0.001).	Providing direct performance feedback via EHR is useful tool to integrate into graduate medical education programs Incorporating direct performance feedback through the EHR is a valuable educational tool and can also improve high risk patient management.
Davis *et al.* ^14^	To determine the effect of EMR template use on residents' documentation of the severity classifications of asthma and appropriate treatment of asthma	Before and after	1. One time 1-hr tutorial on use of asthma template in EHR to document severity and treatment and updated national asthma guidelines. One-on-one tutorials arranged for residents not in attendance. 2. Reminders posted in patient care areas and delivered at daily academic lectures for (6 months	Family Medicine residents / Outpatient practice in a community setting: Spartanburg Family Medicine Residency Program, Spartanburg	EHR data before and after educational intervention	The intervention increased documentation of asthma severity significantly from 24% to 44%. Use of the EMR template significantly increased the rate of inhaled corticosteroid prescriptions, from 36.7% to 71.1%	Use of an asthma template in the EMR improves the knowledge, the documentation of asthma severity and the application of appropriate care.
Stephenson *et al.* ^15^	To determine if training with EHR simulated cases improves learning related to patient safety	Before and after	1 or 2 sessions, at least 1 week apart, consisting of 2 ICU cases in simulated EHR environment with 14 safety issues/action items. Real-time feedback was provided during the session.	Interns, residents, and fellows rotating through the medical Intensive Care Unit in a university setting: Oregon Health and Science University	Data from learner’s performance during simulations before and after educational intervention	Subjects who underwent repeat testing improved in their identification of patient safety issues. Performance improved from 39.9% to 63.6% on the repeat tests.	Engaging in EHR simulation improves trainees’ knowledge and recognition of patient safety issues and enhances their use of EHR systems.
Jyung *et al.* ^16^	To study the effects of (1) implementation of smart phrases in the EHR and (2) an educational session on providers’ knowledge, attitude and use of herbal and dietary supplements (H/DS)	Before and after	One 40-minute teaching session on Integrative Medicine (IM) and tutorial on IM smart phrase use	Mix of residents, attendings, fellows / Outpatient practice in a community and university setting: FQHC and academic health center affiliated with the Icahn School of Medicine at Mount Sinai	1. Survey/ Questionnaire of learners before and after intervention 2. EHR data before and after intervention	The intervention statistically significantly increased participants’ knowledge of the information covered in the IM smart phrases, and increased confidence in using IM smart phrases to start patients on a new supplement. There was no significant change in EHR smart phrase use post-intervention.	To effectively integrate evidence-based use of herbal and dietary supplements in clinical encounters, a sustained educational component is necessary to ensure that EHR smart phrases are a useful strategy.
Long *et al.* ^17^	To assess residents’ cost awareness following sustained visual display of costs into EHR order entry screens	Before and after and survey	8-month display of cost estimates for laboratory orders in EHR	Internal Medicine, Emergency Medicine, Med-peds, OB/gyn, and Orthopedic Surgery residents / Inpatient, outpatient and emergency department in a university setting: Yale-New Haven Hospital	Survey/ Questionnaire of learners before and after intervention	The proportion of residents that agreed or strongly agreed that they knew the costs of tests they ordered increased (8.6% vs 38.2%; p<.0.001). Cost estimation accuracy among residents increased from 24.0% to 52.4% for laboratory orders (p<0.001) and from 37.7% to 49.6% for imaging orders (p<0.001).	Residents’ knowledge of cost of laboratory orders can be significantly enhanced by a comprehensive cost display in the EHR.
Kim *et al.* ^18^	To design and implement a secure messaging competency assessment for residents in a patient-centered medical home (PCMH)	Cross sectional and observation	Development of a standardized rubric by faculty to assess resident competency with secure patient messaging, used during feedback sessions with residents	Family Medicine residents / Outpatient practice in a community-based, urban program affiliated with the University of Washington School of Medicine	EHR data evaluation using standardized assessment tool	Message evaluation showed 10% (n =2) of residents at the novice level, 50% (n = 10) as progressing, and 40% (n = 8) as proficient. The most common deficiencies for residents’ secure messages related to communication rather than clinical competencies (90% vs. 10%). Feedback was integrated into residents’ semiannual evaluations.	Evaluating Family Medicine residents' secure messaging for patient encounters provided valuable feedback on their clinical and communication skills.
Ehrenfeld *et al.* ^19^	To design and implement a performance capture and feedback EHR-based tool that provides data on clinical performance for residents and program directors	Cohort	1. Access to a near real-time, automated, individualized, EHR-based clinical performance feedback tool provided to the resident, duration/ frequency not specified 2. Email with user guide and explanation of tool	Anesthesiology residents / Peri-operative management in a university setting: Vanderbilt University School of Medicine	1. Survey of learners before intervention 2. EHR data after intervention	Overall, >70% residents agreed that they desire frequent updates on their clinical performance on defined quality metrics and how they compare with the residency as a whole. Analysis of the impact of the performance feedback tool on actual resident performance was not completed.	The implementation of near–real-time feedback on resident performance related to specific quality metrics, and modifiable per individual, was positively received by the residents and requires further study to evaluate its effectiveness.
Sutter, Magee ^20^	To study whether incorporating patient counseling prompts into the prenatal EMR increases confidence in and knowledge of counseling	Before and after	28 prenatal topic counseling prompts integrated into EMR for real-time use clinic encounters	Family Medicine residents / Outpatient practice in a community setting: Family health center, Pawtucket, Rhode Island	1. Survey of learners before and after intervention 2. Multiple Choice Exams before and after intervention	Resident average confidence levels and average test scores both increased after prompt use, but the results did not reach statistical significance.	Counseling prompts in the EMR hold promise for enhancing patient education, but further research is needed to determine their effectiveness as an educational tool for residents.
Sperl-Hillen *et al.* ^21^	To develop and implement an innovative virtual model to teach residents the cognitive skills of type 1 and type 2 diabetes management	Before and after	A diabetes educational activity consisting of (a) a curriculum using 18 virtual cases, (b) a web-based interactive interface, (c) a simulation model to calculate physiologic outcomes of resident actions, and (d) a library of programmed feedback to critique and guide resident actions between virtual encounters	Family Medicine, Internal Medicine, Med-peds, Dermatology, Psychiatry, and other residents Outpatient university and community practices	Survey of learners before and after intervention	Mean ratings of knowledge (on a five-point scale) post educational activity improved by +0.5 (p < .01) for use of all available drug classes, +0.9 (p < .01) for how to start and adjust insulin, +0.8 (p < .01) for interpreting blood glucose values, +0.8 (p < .01) for individualizing treatment goals, and +0.7 (p < .01) for confidence in managing diabetes patients.	A virtual educational activity aimed at teaching clinical skills for diabetes management to primary care residents was successfully created, executed, and well-received. It significantly increased residents' self-assessed knowledge and confidence in managing diabetes.
Kurtzman *et al.* ^22^	To measure resident engagement with an EMR-based dashboard providing feedback on their use of routine laboratory tests relative to service averages	Cohort	1. One email to all participants describing the study 2. An additional email to the intervention arm with a snapshot of the dashboard, a link to the online dashboard, and resident and service utilization information 3. Additional follow-up email 24 hours later 4. 3-hour focus groups following completion of the intervention	Internal Medicine residents / Inpatient practice in a university setting: University of Pennsylvania	1. Email read receipts 2. Dashboard engagement via web-based tracking program 3. EHR data before and after intervention 4. Transcripts of focus groups	74% of residents opened the email with link to the dashboard and 21% accessed the dashboard. Residents who opened the dashboard ordered 0.26 fewer labs per doctor-patient-day than those who did not, though the difference was not statistically significant. Themes from the focus groups included that the laboratory dashboard was useful overall, barriers to using the data were that it was too simplistic and did not account for patient complexity, and suggestions for improvement were to give additional guidance on how to use it to change practice and include attendings in the feedback.	Resident engagement with the dashboard overall was low. Certain subsets of residents may be more likely to engage with EMR-based dashboards for practice-based learning. Resident feedback is crucial to tailor dashboard content to meet their educational needs and impact performance.
Hafez, Holhaus ^18^	To improve resident compliance with CMS sepsis core measure documentation through stimulated cases in the EMR	Prospective cohort	1. A brief tutorial on CMS measures: SEP-1, Early Management Bundle, Severe Sepsis and Septic Shock 2. Presentation of 4 clinical vignettes (SIRS without infection, severe Sepsis, 2 septic shock on power point slides)	Emergency Medicine residents / Emergency department in a university setting: Washington University School of Medicine	EHR (in training mode) data before and after the intervention	Resident performance on medication ordering, reassessment and diagnosis was evaluated. The total time required to meet CMS quality measures improved significantly from case 1 (377 +/- 88 sec) to case 4 (173 +/- 37 sec), p=0.001. Resident accuracy improved from 71% in case 1 to 100% in case 4, p<0.001. Repetition of shock cases 3 and 4 showed trends toward improved accuracy and time, but these were not statistically significant.	A 4-case clinical vignette EHR simulation improved residents' efficiency and accuracy in meeting CMS sepsis quality metric documentation. This type of simulation could improve CMS documentation compliance, patient care, and hospital reimbursement.
Sharpe Jr *et al.* ^23^	To increase teaching moments by creating an electronic method for retrieving and displaying changes made to resident-written preliminary reports by attending radiologists	Prospective cohort and survey	An EHR-based web Report Comparator application presenting radiology residents with their 100 most recent preliminary and final report pairs, both side by side and in a “track changes” mode	Radiology residents / Radiology department in a university setting: Thomas Jefferson University Hospital	1. Data on usage of the Report Comparator application 2. Survey of learners before and 6 months after initiation of the intervention	Each resident logged on to the application an average of 16 times over 8 months. Survey responses showed that report comparison increased to ‘daily to weekly’ by most residents (81%) after the intervention. Residents compared reports for educational value, to improve quality of future reports and to improve patient care. They mostly detected edits that were stylistic, helped improve future reports and improved radiology knowledge.	An EHR-based application showing attending edits to preliminary radiology reports with a "track changes" feature was well-liked, and residents perceived that it improved their radiology reporting skills and knowledge.
Hadley Strout *et al.* ^24^	To evaluate a self-directed learning (SDL) based panel management (PM) curriculum	Single group, before and after	1. A panel management curriculum incorporating self-directed learning, EHR-based performance feedback, mentorship from faculty, and goal setting 2. 8 clinic weeks with a 30-min precepted session with faculty and a half day for self-directed learning	Internal Medicine residents / Outpatient practice in a university setting: University of Vermont Medical Center	1. Survey/ Questionnaire of learners before, during and after intervention 2. Focus group after intervention 3. EHR data before, during and after intervention	Post-intervention surveys showed residents found the dashboard useful and likely benefited patients’ health. Focus groups revealed it improved their sense of panel ownership (49%). Barriers identified were lack of continuity with patient panel, and care gap reports not representing residents’ efforts. There was not a statistically significant change in the rate of care gap closure after the intervention.	EHR tools, goal setting, protected time and mentorship in a panel management curriculum were well-received by residents and enhanced patient ownership, but they seek performance feedback that is accurate, timely and easy to interpret. Further investigation is needed on how to prepare residents for future practice and impact patient outcomes.
Shirazian *et al.* ^25^	To determine whether a computerized inpatient renal template note improved resident knowledge and documentation of kidney disease	Before and after	1. A computerized renal template note with clinical decision support was implemented and used for one month. 2. A 30-minute tutorial on electronic note documentation including use of the renal template note and the importance of renal failure documentation 3. One reminder was sent to residents not using the renal template note.	Internal Medicine residents / Inpatient practice in a university setting: Winthrop University Hospital	1. Knowledge quiz of learners before and after intervention 2. Survey of learners before and after intervention 3. EHR data before and after intervention	A significantly higher percent of residents correctly staged earlier stage CKD (CKD stage 3) using the renal template note compared to historical notes (9/46 vs. 0/33, p<0.01). Documentation of AKI and more advanced CKD stages (CKD stages 4 and 5) did not improve. Knowledge quiz scores increased but the change was not statistically significant.	The renal template note significantly improved documentation of early stage CKD, with a modest but non-significant improvement in resident knowledge of kidney disease. Given the importance of early recognition and treatment of CKD, future studies should focus on using template notes in addition to other educational interventions.
March *et al.* ^26^	To determine the frequency of error recognition in an EHR simulation in an ICU setting	Prospective cohort	1. Trainees individually reviewed the chart of a 5-day simulated ICU case within the EHR. 2. Trainees presented the case to a study team member with action items that should be addressed. The study team member also observed the number and type of screens used in reviewing the patient chart. 3. Feedback was provided to trainees immediately after simulation.	Internal Medicine residents, critical care fellows (of pulmonary, medical, and anesthesia background) / Intensive Care Unit in a university setting: Oregon Health and Science University	Observation data from EHR simulation and presentation	The average error recognition rate was 41% (range 6–73%), which increased with the level of training (35%, 41% and 50% for interns, residents, and fellows, respectively). Over-sedation was the least-recognized error (16%); poor glycemic control was most often recognized (68%). Only 29% of trainees recognized changes in vital signs consistent with recurrent sepsis.	Since significant gaps remain in identifying medical management errors by physicians within comprehensive EHRs, EHR-specific training with tools such as simulation may be beneficial to identify these gaps and ultimately improve patient care.
Sebok-Syer *et al.* ^27^	To investigate how EHR data can be used to assess residents’ independent and interdependent clinical performance and how performance can be represented in a resident report card	Single group (one time observation/intervention)	1. One time 30-45minute semi-structured individual interview with 10 Emergency Medicine (EM) faculty and 11 EM residents (all years) 2. Open-ended questions about a) the utility of a list of EM practice metrics measurable in the EHR in assessing resident performance and b) the extent to which each metric reflects independent or interdependent performance	Emergency Medicine residents and faculty / Emergency Department in a university setting: Stanford University School of Medicine	Semi structured interviews	An Emergency Medicine assessment tool, including a list of 12 metrics, was developed based on EHR data and discussion with both clinical faculty and residents. Metrics were labeled as representative of resident independence or interdependence (eg. 94% of participants responded that “turnaround time between signup and first orders” reflected independence).	This systematic approach to developing EM resident assessments integrates the viewpoints of both clinical faculty and residents on EHR data, enhancing understanding of clinical performance and differentiating between independent and interdependent measures in collaborative, workplace settings.
Tisdale *et al.* ^28^	To measure the effect of an EMR-based handoff tool on resident handoff completeness	Before and after	1. Implementation of an EMR handoff template based on the I-PASS framework 2. Introduction of this tool at resident educational conferences and intern orientation 3. Handoff tool was displayed on the EMR landing page for all providers and nurses	Internal Medicine residents / Inpatient practice in a university setting: Stanford University School of Medicine	1. EHR data before and after intervention 2. Survey/ Questionnaire of nursing staff working with trainees	The proportion of handoffs that included medications, laboratory tests, vital signs and assessment of illness severity increased significantly between pre- and post-implementation. Illness severity reporting increased by 97.8% and inclusion of vitals increased by 81.6%. Nursing surveys showed most nurses used the handoff report, found it helpful, and paged the on-call MD team less often as a result.	This study demonstrates that EMR-based interventions could enhance the handoff communication process, potentially resulting in fewer medical errors and improved patient care.

## Results

All included studies were set in North America. Forty-seven percent (9) of the studies took place in the inpatient setting, 37% (7) in outpatient setting, 11% (2) in the emergency room setting, and one study took place at all three sites. Thirty-two percent (6) of the studies were in internal medicine programs, 21% (4) in family medicine, 11% (2) in emergency medicine, 11% (2) in anesthesia, 5% (1) in radiology, and the rest involved multiple specialties. Sixteen studies involved participants in residency training while three also included fellows. Thirteen studies were designed to gather data before and after the intervention. Six were cohort studies ( Table 1).

The four main themes of the educational interventions were: 1. Utilization of the EHR for assessment and performance feedback, 2. Using EHR simulation to teach, 3. Using the EHR to impart clinical knowledge, and 4. Using the EHR to teach systems-based practice. 

### 1. Utilization of the EHR for assessment and performance feedback

The EHR can be used for assessment and performance feedback through tracking of patient care metrics, procedure logs, documentation quality, and productivity. Our scoping review found six studies that used the EHR purposefully for teaching and learning through assessment and performance feedback.

Green *et al.* aimed to improve anesthesiology residents’ adherence to evidence-based post-operative management of nausea and vomiting ^13^. Thirteen first-year anesthesiology residents participated in a teaching intervention that consisted of a 15-minute lecture, followed by two snapshots of data gathered via the EHR on individual compliance to the guidelines. This feedback significantly increased residents’ compliance with post-operative nausea and vomiting prophylactic treatment guidelines. Most residents (70%) found the feedback helpful for their learning, and most (80%) were interested in seeing how their metrics compared with their co-residents.

Ehrenfeld *et al.* also focused on feedback for anesthesiology residents, but in the setting of perioperative management ^19^. Around 24,000 cases completed by 60 anesthesiology residents were assessed on the following five quality metrics: antibiotics administration, glucose monitoring, central line insertion, pain management, and temperature management. They were provided feedback on the real-time data along with explanations which served as teaching moments. Residents reported that they learned from the interaction and desired frequent feedback on their clinical performance.

Kim *et al.* used the EHR to develop a teaching tool to assess family medicine residents’ clinical and communication competency through evaluation of secure patient message encounters in the EHR ^18^. Portal messages from ten residents revealed that the most common deficiency was related to communication errors (90%) rather than clinical competency (10%). Some examples of communication errors were late replies to patients, lack of empathetic tone, and use of excessive medical jargon. Faculty used the tool to teach and provide feedback, which was incorporated into residents’ semi-annual evaluations.

Sharpe Jr. *et al.* created an EHR teaching tool to provide feedback on radiology residents’ imaging reports ^23^. The tool captured changes made to resident’s preliminary reports by attendings. Over an eight-month period, 65 residents viewed 4,408 reports to track changes. Surveyed residents reported they used this tool for education, to improve their future reports, and to improve patient care.

Hadley Strout *et al.* created a panel management curriculum for 35 internal medicine residents that incorporated self-directed learning (SDL), EHR-driven performance feedback, and mentorship for setting learning and patient care goals ^24^. They used the SDL model to uncover knowledge gaps, gain and apply knowledge, and reflect on learning ^29^. Residents participated in 30-minute sessions with faculty to reflect on their care gap report and set goals, such as screening for social determinants of health and advance directive completion. Data was gathered from pre- and post-intervention surveys, weekly surveys, and focus groups. Though results did not show any significant differences between the pre- and post-intervention period, residents valued the dashboard data, mentorship, autonomy to set learning and care gap goals, and increased sense of panel ownership.

Sebok-Seyer *et al.* tracked the performance of residents in the emergency room with data entered into the EHR, followed by 30-minute interviews with ten emergency medicine faculty and 11 emergency medicine residents to investigate how various metrics can be used to teach and assess clinical performance ^27^. The interviews invited reflections from the faculty and trainees, who discussed the importance of the metrics, challenges with interpretation, and the differences between measures that reflect a resident’s independence versus measures that may reflect multiple interdependent factors in a setting like the Emergency Department. They generated a list of 12 metrics that should be used for EM resident assessments, some of which included number of patients seen, time between signing up for a new patient and entering first orders, and time to antibiotics.

### 2. Using EHR simulation to teach

Simulation in the EHR is a powerful tool for training residents on real-world scenarios, order entry, prioritization, error management, and compliance with healthcare regulations. Our scoping review found four studies that incorporated EHR simulation. Lee *et al.* used a simulated EHR clinical vignette to teach 76 internal medicine residents about appropriate ICD-10 documentation ^11^. The intervention consisted of a three-part team-based learning session which incorporated a simulated EHR and required selection of appropriate ICD-10 code diagnoses. As an example, residents were asked to go to the order entry screen of the EHR, order tests, and associate each with an appropriate diagnosis code. The pre- and post-testing demonstrated an increase in knowledge about coding.

March *et al.* and Stephensen *et al.* used a novel EHR simulation to identify patient safety issues in the ICU related to labs, vital signs, medication administration, ventilator settings, nursing and notes. Fellows and residents received feedback after presenting the simulated cases ^15, 26^. In the second study by Stephensen *et al.*, correct identification of patient safety issues via the EHR simulation improved on the second test with a different simulated ICU case, suggesting that repeated simulation enhances learning.

Hafez *et al.* used simulated cases to improve emergency medicine resident’s compliance with Centers for Medicare and Medicaid Services (CMS) sepsis core measures ^30^. The intervention consisted of an educational session on quality measures, followed by using the EHR in training mode to assess proper medication administration, reassessment, and final diagnosis for four clinical vignettes. The residents significantly improved from case one to case four in both the time required to meet CMS quality measures and diagnostic accuracy.

### 3. Using EHR to impart clinical knowledge

The EHR can be used to teach numerous clinical concepts through modules, documentation templates, and smart phrases. Smith *et al.* used a web-based teaching EHR to improve senior internal medicine and medicine-pediatrics residents’ ability to make a working diagnosis and enter admission orders for patients in the ICU setting ^12^. Three modules were created using real patient data with the diagnosis of severe sepsis or septic shock. Residents were provided with immediate feedback after each module. Smith *et al.* found that residents who completed all three modules improved their diagnostic accuracy from 45% to 81%. 85% of residents thought the modules were an effective way to teach.

Davis *et al.* investigated whether family medicine residents’ use of an EHR template for documentation of asthma severity classification would lead to improved treatment ^14^. The intervention included a one-hour lecture on use of the template and presented updated asthma guidelines. A pre- and post-intervention chart review of 180 patients seen by the residents found that use of the template significantly improved documentation of asthma severity from 24% to 44% and increased the rate of inhaled corticosteroid prescriptions from 37% to 71%.

Jyung *et al.* studied the effect of using smart phrases in the EHR on knowledge of, attitudes toward, and use of herbal and dietary supplements ^16^. Participants included mostly residents, as well as attendings and fellows. The intervention was a 40-minute teaching session that included information on five herbal and dietary supplements and a tutorial on the use of the EHR smart phrase. The improvement in knowledge post-intervention did not reach statistical significance. While participants noted increased confidence in starting a new supplement, there was no increase in the actual frequency of smart phrase use.

Sutter *et al.* examined whether using prenatal counseling prompts integrated into the EHR increased counseling knowledge and confidence in second- and third-year family medicine residents ^20^. Detailed prompts on 28 prenatal counseling topics were created. Six months after release of the prompts, a survey and a multiple-choice knowledge test revealed higher confidence levels and higher test scores, but the results did not reach statistical significance.

Sperl-Hillen *et al.* aimed to improve management of type 1 and type 2 diabetes through 18 virtual cases, a web-based EHR interactive interface, a simulation model to predict physiologic outcomes, and feedback ^21^. Residents received three new cases monthly, which could be completed multiple times. Residents achieved mastery in a case if they reached predetermined clinical targets for A1c, blood pressure, and low-density lipoprotein cholesterol without patient safety errors. Pre- and post-education resident surveys using a five-point Likert scale showed improvement in reported knowledge in using all available drug classes, initiating and adjusting insulin, interpreting glucose values, and overall confidence in managing patients with diabetes.

Shirazian *et al.* sought to determine whether a renal template note would improve internal medicine residents’ knowledge and documentation of kidney disease ^25^. The intervention consisted of a tutorial on the EHR note template, a lesson on the importance of documentation of kidney disease, an fill-in-the-blank knowledge test, and a reminder to residents not using the template. Progress notes using the template were compared to historical progress notes. Shirazian *et al.* found that documentation of stage 3 CKD improved significantly. There was improvement in documentation of acute kidney injury and chronic kidney disease (CKD) stage 4–5, as well as improvement in scores on the knowledge test, but these did not reach statistical significance.

Tisdale *et al.* studied the effect of using an EHR-based hand-off template to teach about hand-off completeness for internal medicine residents ^28^. Prior to the intervention, the hand-off was entirely free text, and the intervention consisted of a template based on I-PASS elements such as a to-do list, contingency plan, and illness severity evaluation. Among the three elements that showed statistically significant improvement in completion rate (labs, vitals, and illness severity), illness severity was considered the most crucial given its importance for communicating a concise description of a patient’s clinical status.

### 4. Using the EHR to teach systems-based practice

The EHR can teach systems-based practice through review of resource utilization, cost awareness, patient safety, system errors, and performance on care quality measures. Long *et al.* evaluated and provided feedback about resident physicians’ cost awareness of laboratory and imaging orders ^17^, while Kurtzman *et al.* studied laboratory utilization of residents ^22^. Long’s intervention involved displaying the costs for 1032 laboratory orders to internal medicine, pediatrics, obstetrics and gynecology, emergency medicine, and orthopedic surgery residents. In addition to administration of a pre- and post-intervention survey, residents were asked to estimate Medicare reimbursement for 11 commonly ordered laboratory and imaging tests. The number of residents who agreed that they knew the costs of tests they ordered improved significantly. Furthermore, improvement of cost estimation for laboratory and imaging orders achieved statistical significance. Kurtzman’s intervention consisted of creating a dashboard that displayed the average number of labs per patient day ordered by each resident and using the dashboard to provide feedback to the residents. Residents’ utilization of the dashboard was measured using a web-based tracking platform. The study did not find a statistically significant difference in laboratory orders by residents who did versus did not use the dashboard.

## Discussion

Our study reviewed nineteen studies that purposefully used the EHR in educational interventions for teaching and learning with resident trainees. In these studies, the main educational functions of the EHR were to: assess performance and provide feedback, teach via simulation, impart clinical knowledge, and teach systems-based practice. One of the key findings of our study is that very few researchers intentionally set out to utilize the EHR for the purpose of teaching residents and to assess its impact on their learning.

As the EHR has become the all-encompassing clinical platform used by learners to deliver healthcare to patients throughout their training, there is a relative gap in the literature in the description of how the EHR is being used purposefully by teachers, faculty and program leadership to advance trainees’ clinical skills and measure their progress. Additional purposefully designed educational intervention studies are needed to understand the EHR-based strategies that effectively teach clinical and systems-based skills, lead to sustained learning, and engage trainees in a way that they will carry into their future practice. We propose that residency program directors, educational leaders and faculty incorporate teaching and learning processes into the EHR while being cautious of limiting this to best practice alerts and reminders, as these features can improve patient outcomes but do not necessarily promote deeper understanding in trainees. Furthermore, training programs should consider assigning a role for an EHR education lead who would work with IT teams who drive upgrades and clinical optimizations of the system to incorporate changes needed for teaching and learning.

## Limitations

To our knowledge, this is the first scoping review to evaluate uses of the EHR explicitly for teaching and learning purposes in graduate medical education. However, there are limitations to our review. Some studies may not have clearly articulated an educational intervention goal but could have included a component that was not described in the abstract. Non-English language studies were excluded, which may have limited our review of relevant studies from other countries. Lastly, we excluded studies performed with other learners including medical students, which is a larger population of learners, and therefore may have missed studies with strong examples of teaching and learning clinical skills via the EHR.

## Conclusion

There are relatively few studies that purposefully utilize the EHR as a teaching and learning tool in educational interventions with resident trainees. Where it is employed, it is used in simulation, for assessment and performance feedback, in teaching clinical knowledge and skills, and in teaching systems-based practice, with positive impacts on learning and positive experiences by trainees. However, further study is warranted to examine EHR-based teaching strategies that improve trainees’ skills and knowledge without causing undue burden.

## Ethics and consent

Ethical approval and consent were not required.

## Data Availability

No data are associated with this article. **Figshare:** The electronic health record as a teaching and learning tool in residency training: A scoping review. https://doi.org/10.6084/m9.figshare.28829981.v3 ^31^. This project contains the following extended data: Appendix 1 MeSH Terms for database search Data Extraction Sheet Figure 1. PRISMA diagram Data are available under the terms of the Creative Commons Attribution 4.0 International license (CC-BY 4.0). ***Figshare:*** PRISMA-Scr checklist for “The electronic health record as a teaching and learning tool in residency training: A scoping review”. https://doi.org/10.6084/m9.figshare.28829981.v3 ^31^ Data are available under the terms of the Creative Commons Attribution 4.0 International license (CC-BY 4.0).
