# Supplementary material for: The electronic health record as a teaching and learning tool in residency training: A scoping review
Source: MedEdPublish (2016). 2025 Aug 7;15:21. Originally published 2025 May 7. [Version 2] doi: 10.12688/mep.20947.2 (PMC13684029; doi:10.12688/mep.20947.2)
Supplement: Peer review history

# Reviewer report: Reviewer response for version 2

**DOI:** 10.21956/mep.22740.r42937

**Published:** 2025-09-05

**Authors:** Augustino Mwogosi

**Affiliations:** 1 The University of Dodoma, Dodoma, Dodoma Region, Tanzania

This scoping review, conducted by Rubin et al., investigates how the electronic health record (EHR) is used as a teaching and learning tool in graduate medical education. The authors state that while EHRs are widely used in clinical care, there is a gap in the literature regarding their purposeful use in educational interventions. The review followed the Arksey and O'Malley framework and the PRISMA-ScR guidelines. A search of multiple databases (PubMed, SCOPUS, CINAHL, ERIC, Cochrane, and MedEdPORTAL) from 2010 to 2022 yielded 19 studies that met the inclusion criteria.

 The review found four main themes for educational interventions using the EHR:

1. Assessment and performance feedback.
2. EHR simulation to teach.
3. Using the EHR to impart clinical knowledge.
4. Using the EHR to teach systems-based practice.

The authors conclude that the EHR is being used as a teaching tool, but more purposeful use is needed in designing educational interventions for residents.

 The rationale for the review is clearly stated in the introduction, highlighting the widespread adoption of EHRs in healthcare and the need for new approaches to integrate this technology into medical education. The authors explicitly state a gap in the literature regarding the purposeful use of EHRs for teaching and learning outside of efficiency training or quality improvement projects.

 The objective is presented as a specific research question: "How is the EHR purposefully used in educational interventions for teaching and learning in graduate medical education?" This question serves as the clear objective for the review.

 While the authors provide a good overview of their methodology, some crucial details are missing, which would hinder replication.

**Constructive and Specific Details for Authors to Address:**

1. **Search Strategy:** The article mentions that a search was conducted using keywords and MeSH terms but does not provide the exact search strategy. This is a critical omission for a systematic review. To make the article scientifically sound, the authors **must** provide the full search strategy for each database, either in the main text or in an appendix. This is the single most important point to address for reproducibility.
2. **Study Selection Process:** The authors mention using Covidence® for screening. While they state that three reviewers independently screened full-text articles and resolved conflicts, they do not report the inter-rater reliability, such as a Kappa score. Reporting this metric is standard practice for systematic reviews to demonstrate the rigor of the screening process.
3. **Data Extraction:** The article states that a data extraction form was developed a priori and refined after pilot testing, but it does not provide the form itself or its full contents. Providing a copy of the data extraction form, or a detailed description of all fields, would be beneficial for replication.

The conclusions of the review are well-supported by the results presented. The authors' conclusion that the EHR is being used as a teaching tool is directly supported by the descriptive analysis of the 19 included studies, which outlines four main thematic uses. The conclusion that there is a need for more purposeful use of the EHR is a logical extension of their findings that a relatively small number of studies met their criteria for having a "purposeful design for an educational intervention." The provided Table 1, which details the interventions and outcomes of the included studies, serves as the direct evidence for the conclusions. The paper accurately reflects the findings of the included studies and correctly presents a need for further research in the area.

Are the rationale for, and objectives of, the Systematic Review clearly stated?

Yes

Is the statistical analysis and its interpretation appropriate?

Not applicable

Are sufficient details of the methods and analysis provided to allow replication by others?

Partly

Are the conclusions drawn adequately supported by the results presented in the review?

Yes

Reviewer Expertise:

Health informatics

I confirm that I have read this submission and believe that I have an appropriate level of expertise to confirm that it is of an acceptable scientific standard, however I have significant reservations, as outlined above.

# Reviewer report: Reviewer response for version 1

**DOI:** 10.21956/mep.22432.r41918

**Published:** 2025-06-09

**Authors:** Jesse Burk-Rafel

**Affiliations:** 1 New York University Grossman School of Medicine, New York, New York, USA

This manuscript addresses an important gap in the literature: the use of data/insights from the EHR for GME education. A scoping review is the appropriate method to address the gap of of what is known on this topic. 

 I found the paper to be exceptionally well written and clear. The authors screened a large number of papers; I feel the scope of the paper and exclusion criteria are reasonable and still address an important area with a dearth of published work. 

 The identified studies were diverse and led the authors to 4 key themes that inform work in this space, including the limitations of EHR-based audit-and-feedback. I appreciated greatly the summary table with key findings, including limitations. The scale and density of feedback afforded by leveraging the EHR is clear from this scoping review.

 The results indicate the published literature is limited to single site, limited power studies, pre-post within subject design without randomization or crossover, assessment of low-level Miller taxonomy outcomes.

 Space permitting, this paper would be most strengthened through an expansion of the discussion -- the results are clearly stated, but "So what? Now What?". What questions are well answered by the identified studies, and what major gaps in the literature remain? Can you delineate some areas that the field should move towards?

Are the rationale for, and objectives of, the Systematic Review clearly stated?

Yes

Is the statistical analysis and its interpretation appropriate?

Yes

Are sufficient details of the methods and analysis provided to allow replication by others?

Yes

Are the conclusions drawn adequately supported by the results presented in the review?

Yes

Reviewer Expertise:

Precision education research, including the use of the EHR for educational purposes. I am not a secondary research expert, however, so would defer to other reviewers on specific methods used in the gathering and screening the records, though they appear to follow PRISMA guidelines.

I confirm that I have read this submission and believe that I have an appropriate level of expertise to confirm that it is of an acceptable scientific standard, however I have significant reservations, as outlined above.
